# Age Is a Critical Risk Factor for Severe Fever with Thrombocytopenia Syndrome

**DOI:** 10.1371/journal.pone.0111736

**Published:** 2014-11-04

**Authors:** Shujun Ding, Guoyu Niu, Xuehua Xu, Jinping Li, Xiaomei Zhang, Haiying Yin, Naijie Zhang, Xiaolin Jiang, Shiwen Wang, Mifang Liang, Xianjun Wang, Xue-jie Yu

**Affiliations:** 1 School of Public Health, Shandong University, Jinan, Shandong Province, China; 2 Department of Viral Infectious Diseases Control and Prevention, Shandong Center for Disease Control and Prevention, Shandong Provincial Key Laboratory of Communicable Disease Control and Prevention, Jinan, Shandong Province, China; 3 Key Laboratory for Medical Virology, MOH, National Institute for Viral Disease Control and Prevention, China CDC, Beijing, China; 4 Tianjin International Travel Health Care Center, Tianjin, China; 5 Department for communicable Diseases Control and Prevention, Laizhou City Center for Disease Control and Prevention, Laizhou, Shandong Province, China; 6 Department of Pathology, University of Texas Medical Branch, Galveston, Texas, United States of America; German Primate Center, Germany

## Abstract

**Background:**

Severe Fever with Thrombocytopenia Syndrome (SFTS) is an emerging infectious disease in East Asia. SFTS is a tick borne hemorrhagic fever caused by SFTSV, a new bunyavirus named after the syndrome. We investigated the epidemiology of SFTS in Laizhou County, Shandong Province, China.

**Methods:**

We collected serum specimens of all patients who were clinically diagnosed as suspected SFTS cases in 2010 and 2011 in Laizhou County. The patients' serum specimens were tested for SFTSV by real time fluorescence quantitative PCR (RT-qPCR). We collected 1,060 serum specimens from healthy human volunteers by random sampling in Laizhou County in 2011. Healthy persons' serum specimens were tested for specific SFTSV IgG antibody by ELISA.

**Results:**

71 SFTS cases were diagnosed in Laizhou County in 2010 and 2011, which resulted in the incidence rate of 4.1/100,000 annually. The patients ranged from 15 years old to 87 years old and the median age of the patients were 59 years old. The incidence rate of SFTS was significantly higher in patients over 40 years old and fatal cases only occurred in patients over 50 years old. 3.3% (35/1,060) of healthy people were positive to SFTSV IgG antibody. The SFTSV antibody positive rate was not significantly different among people at different age groups.

**Conclusion:**

Our results revealed that seroprevalence of SFTSV in healthy people in Laizhou County was not significantly different among age groups, but SFTS patients were mainly elderly people, suggesting that age is the critical risk factor or determinant for SFTS morbidity and mortality.

## Introduction

Severe fever with thrombocytopenia syndrome (SFTS) is an emerging hemorrhagic fever in East Asia. The disease is caused by SFTSV, a phlebovirus in *Bunyaviridae* family [1]. SFTS had been found for the first time in China in 2010 [1] and it was reported in Korea and Japan in 2013 [2], [3]. SFTSV had a high case fatality, which was 12–30% in China [1].The annual incidence rate of SFTS was not well documented in China. One report showed that in 2011 and 2012, 2,047 cases of SFTS were reported in China [4]. The major clinical symptoms and laboratory abnormalities of SFTS were non-specific, including fever, thrombocytopenia, leucopenia, and elevated serum hepatic enzymes [1], [5]. SFTSV was most likely to be transmitted by tick bite because the virus was detected from *Haemaphysalis longicornis* ticks [1]. Person to person transmission of SFTS through contact with infected patient's blood or mucous also occurred in hospitals in China [6]–[8]. Animal host of SFTSV included domestic animals such as goats, dogs, cattle, pigs and chickens and small mammals such as rodent and shrews [5], [9]–[11]. Although several previous studies have recognized that only old people got hospitalized and died of SFTSV [1], [4], [12]–[16], there was no systemic comparison of the SFTSV infection rate in different age groups. In this study we investigated the seroprevalence of SFTSV in different age groups and the clinical cases of SFTS in Laizhou County, Shandong Province in China. We found that age was the critical risk factor or determinant for SFTSV morbidity and mortality.

## Methods

### Ethics statement

The study was approved by the Ethic Committee of Preventive Medicine of Shandong Center for Disease Control and Prevention (Shandong CDC). All study objects had signed an informed consent document prior to participation. A signed informed consent was required from their parents or legal guardian for minors. All data analyzed were anonymized.

### Study site

Laizhou County located in east longitude 119° 33′ and 120° 18′ and between north latitude 36° 59′ and 37° 28′. It had a total area of 1, 878 square kilometers and a total population of 858,750 and a rural population of 493,100 on average in 2010–2011. Laizhou County had a sub-humid northern temperate monsoon continental climate and annual rainfall of about 610 mm. The annual average temperature was about 12 degrees Celsius. There were hills and mountains in the southeast and plains in the northwest. In Laizhou, the area was 10.3% low mountains, 48.1% hills, and 41.6% plains.

### Serum samples of healthy persons

Among the villages that had reported SFTS cases, we randomly selected 30 villages as target villages to collect healthy persons' serum specimens. In these selected villages, people were divided into 8 age groups as 0, 10, 20, 30, 40, 50, 60, and ≥70. In each group 50–100 persons were randomly selected to collect serum specimens in June in 2011. For the survey, healthy persons were people who had never been hospitalized because of illness resembling SFTS in the past. The healthy persons might not completely exclude persons, who had mild SFTS, but never hospitalized. However, they should not be major population in the healthy persons because the incidence rate of SFTS was very low [17].The study was approved by the Ethic Committee of Preventive Medicine of Shandong Center for Disease Control and Prevention (Shandong CDC). All sampled persons had signed an informed consent document. 5 ml of blood was drawn from each person and shipped to the Laizhou CDC on ice. Serum was separated by centrifugation and frozen at −80°C until use.

### Serological test

Sera were tested for SFTSV specific IgG antibody using indirect ELISA described previously [18]. The experiment included negative control, positive control and blank control. To each well 100 µl of a sample (10 µl serum dilute in 90 µl sample diluent) was added except for control wells, and then incubated at 37°C for 30 minutes. After washing five times, each well was added 100 µl horse radish peroxidase (HRP) labeled reagent, then the plate was incubated at 37°C for 30 minutes. After washing five times, a chromogenic agent A and B solution were added to each well to develop the color and the plate was read at 450 nm for optical density (OD). The OD value of a sample ≥ threshold value (cutoff) was considered as positive. The threshold value  = 0.10 + the average OD value of the negative control (if the OD value of a negative control was less than 0.04, it was considered as 0.04).

### Statistical analysis

Statistical analysis was performed using SPSS18.0 software and P<0.05 was considered as statistically significant difference.

### Real-time Fluorescence Quantitative PCR (RT-qPCR)

The total RNA of every clinical patient's serum specimen was extracted using the QIAamp Viral RNA Mini Kit, and RNA was used as template for qPCR to amplify SFTSV S, M, and L segments of SFTSV RNA using primers described previously [19].

### SFTS case definition

The definition of suspected SFTS cases was patients with acute fever, thrombocytopenia and/or leucopenia. Acute serum specimens of all suspected SFTS cases reported in 2010 and 2011 in Laizhou County were submitted to the Laizhou County CDC. The laboratory confirmed cases were defined as SFTS patients who were hospitalized and confirmed to be infected with SFTSV by qRT-PCR for at least two segments of L, M and S segments of the viral genome.

## Results

### SFTS patients

82 patients in 2010 and 70 patients in 2011 were reported as suspected SFTS cases, and only 30 patients in 2010 and 41 patients in 2011 were confirmed to be infected with SFTSV by RT-qPCR (Table 1). The case fatality of SFTS was 14% (10/71) in 2010 and in 2011. The ages of the patients ranged from 15 years old to 87 years old and the median age was 59 years old. Male and female ratio of the patients was 1.03∶1. The fatal cases ranged from 51 years old to 81 years old and the median age of fatal case was 74 years old. The incidence rate of persons over 40 years old was 7.1/100,000, and the incidence rate of persons less than 40 years old was 0.5/100,000 in Laizhou. The incidence was significantly different between these two age groups by using Fisher's exact test (p<0.01).There was no death case reported in persons under 50 years old in 2010–2011 in Laizhou. While the mortality rate of persons over 50 years old was 1.6/100,000. There was also a high significant statistic difference of mortality rate between these two age groups by using Fisher's exact test (p<0.01). The incidence rate of SFTS was 4.1/100,000 in Laizhou totally. Because all of the 71 SFTS cases were farmers, according to the Statistic Yearbook of Laizhou and the public information on website (http://baike.baidu.com/subview/9494/7451701.htm?fromtitle=%E8%8E%B1%E5%B7%9E%E5%B8%82&fromid=2405410&type=syn), near 60% (493100/858750) population were farmers in Laizhou, so the incidence rate of SFTS in rural population was 7.2/100,000(35.5/493100) in Laizhou County.

**Table 1:** The age distribution of SFTS cases from 2010 to 2011 in Laizhou.

Age groups	No. of suspected SFTS cases	Laboratory confirmed SFTS cases	SFTS Incidence rates (/100,000)	SFTS fatal cases	Fatality rate %
0-	2	0	0	0	0
10-	4	1	0.6	0	0
20-	7	0	0	0	0
30-	20	3	1.2	0	0
40-	35	12	3.7	0	0
50-	44	20	6.6	3	15
60-	28	18	9.9	1	5.6
≥70	12	17	12.6	6	35.3
Total	152	71	4	10	14.1

In 2011, 30 villages were randomly selected from 39 villages that had SFTS patients for SFTSV seroprevalence investigation. The healthy persons were divided into 8 age groups with every ten years as one group from 0 years old to 69 years old and people who were ≥70 as one group. 1,060 healthy persons were randomly selected with each group 50 to 100 persons. 3.3% (35/1,060) of the healthy persons were serum antibody positive to SFTSV by ELISA (Table 2). The SFTSV antibody positive persons were distributed in all age groups. Seropositive rate was higher in age groups 10 ∼, 30 ∼ and ≥70 than that in other groups, but it was not significantly different among the age groups (χ^2^ = 5.31, P>0.05). 2.3% (12/513) male were serum antibody positive to SFTSV and 4.2% (23/547) female were serum antibody positive to SFTSV. The difference of seropositve rate was not statistically significant between male and female (χ^2^ = 2.89, P>0.05). Seropositive rate was not significant different between students (3.9%, 11/285) and farmers (3.1%, 24/775) (χ^2^ = 0.38, P>0.05) (table 3).

**Table 2:** Seroprevalence of SFTSV in healthy human volunteers in Laizhou.

Age groups	Number of sample	Number Positive	Positive rate %
0-	125	3	2.4
10-	162	8	4.9
20-	100	4	4
30-	113	5	4.4
40-	149	3	2
50-	138	2	1.4
60-	136	4	2.9
70-	137	6	4.4
Total	1060	35	3.3

**Table 3:** Comparison of SFTS IgG in different occupational groups of healthy volunteers in Laizhou.

Occupation	Sampled persons	Number of positive	Positive rate%	?^2^	P
Famers	775	24	3.1	0.38	>0.05
Students	285	11	3.9		

## Discussion

SFTS is an emerging infectious disease and causes a high case fatality. Little was known about the pathogenesis and risk factors of SFTS. Previous studies had demonstrated that SFTS patients ranged from 1 to 90 years old, but the median age of SFTS patients were approximately 60 years old. The high incidence of SFTS in elder people in China was presumed to be caused by the fact that elder people had more opportunity to expose to ticks than young people because elder people lived in rural areas and young people and kids had migrated to cities to work and to go to school. In this study we investigated the SFTS cases and seroprevalence of SFTSV in healthy persons in Laizhou County. We found that all age groups had similar seropositive rate to SFTSV. These results suggested that all age groups in Laizhou had similar exposure opportunity to SFTSV. Our study demonstrated that SFTS patients were predominantly elderly people in Laizhou County. These results suggested that people at all age groups were susceptible to SFTSV infection, but only old people got severe disease and needed to be hospitalized or even died of SFTSV infection. We found that the antibody levels in all age groups were different, but there were not significantly different. To avoid possibility of small subject number in each group, we combined two neighbor groups together and did statistical analysis again, but we still did not find significant difference among the groups. Thus, we concluded that age was a critical risk factor for SFTS morbidity and mortality. A recent study demonstrates that mice was resistant to SFTSV, but alpha/beta interferon receptor (IFNAR^−/−^) knockout mice were highly susceptible to SFTSV infection [20]. It suggested that alpha/beta IFNs were essential in resistance to SFTSV infection. The elder persons might have low immunity to SFTSV and became more susceptible to SFTSV infection.

We demonstrated that the annual incidence of SFTS is 4.1/100,000 in Laizhou County, which was similar to Yiyuan County in Shandong Province, but seroprevalence rate of SFTSV in Laizhou County (3.3%) was higher than that in Yiyuan County (1.0%). The seroprevalence rate of SFTSV in Yiyuan County and Laizhou County might reflect people had different exposures to SFTSV in different places or might be caused by different ELISA assay to detect IgG antibody to SFTSV. We used indirect ELISA and the previous study used double antigen sandwich ELISA. Serosurvey in Jiangsu Province demonstrated that seroprevalence rate of healthy persons was 3.6% [9]. These studies indicated that subclinical and mild SFTSV infection occurred in rural populations in China.

Previous studies were designed to investigate the seroprevalence of SFTSV in total population, which did not differentiate the age distribution of SFTSV antibody positive persons [5], [9], [11]. In this study we differentiated the age distribution of SFTSV antibody positive persons and demonstrated that the distributions of SFTSV antibody positive persons are not significantly different among the age groups. It suggested that all age groups had similar exposure to SFTSV. We also showed that students and farmers who lived in the same rural areas were not significantly different in SFTSV antibody positive rate. This indicated that students and famers in the rural areas of Laizhou had equal opportunity to exposure to SFTSV [1], [10]. SFTSV was presumed to be transmitted by tick bite because ticks collected from animals harbor SFTSV. Whether SFTSV had other vector needed to be further investigated. In Laizhou County, SFTSV virus had been detected from all domestic animals investigated including sheeps, cattle, dogs, pigs and chickens [10].

Our results showed that SFTSV specific IgG antibodies were detected in people of different age groups and seropositive rate was not significantly different among people of different ages, indicating that ages was not a risk factor for SFTSV infection; but SFTS onset and death mainly occurred in the elderly population and the incidence of SFTS with a significant age aggregation, therefore, we concluded that age is the risk factor or determinant for SFTSV morbidity and mortality.
